# Target Coverage and Normal Organ Sparing in Dose-Escalated Total Marrow and Lymphatic Irradiation: A Single-Institution Experience

**DOI:** 10.3389/fonc.2022.946725

**Published:** 2022-07-26

**Authors:** Chunhui Han, An Liu, Jeffrey Y.C. Wong

**Affiliations:** City of Hope Comprehensive Cancer Center, Duarte, CA, United States

**Keywords:** helical tomotherapy, VMAT, acute lymphoid leukemia, hematopoietic stem cell transplant, total marrow and lymphatic irradiation

## Abstract

**Purpose/Objectives:**

The aim of this study is to report historical treatment planning experience at our institution for patients receiving total marrow and lymphatic irradiation (TMLI) as part of the conditioning regimen prior to hematopoietic stem cell transplant.

**Materials/Methods:**

Based on a review of all historical clinical TMLI treatments plans, we retrieved a 12-Gy cohort of 108 patients with a prescription dose of 12 Gy to the skeletal bones, lymph nodes, spleen, and spinal canal, and retrieved a 20-Gy cohort of 120 patients with an escalated prescription dose of 20 Gy to the skeletal bones, lymph nodes, spleen, and spinal cord, and 12 Gy to the brain and liver. Representative dosimetric parameters including mean and median dose, D80, and D10 (dose covering 80% and 10% of the structure volume, respectively) for targets and normal organs were extracted and compared between the two groups of patients.

**Results:**

For the 12-Gy cohort, the average mean dose for normal organs ranged from 18.3% to 78.3% of 12 Gy, and the average median dose ranged from 18.3% to 77.5% of 12 Gy. For the 20-Gy cohort, the average mean dose for normal organs ranged from 13.0% to 76.0% of 20 Gy, and the average median dose ranged from 12.5% to 75.0% of 20 Gy. Compared to the mean dose to normal organs in the 12-Gy cohort, the average mean dose to normal organs increased from 0.0% to 73.1%, with only four normal organs showing a >50% increase. Normal organ dose in TMLI plans using volumetric modulated arc therapy fields fell within the dose range in historical TMLI plans.

**Conclusion:**

Dosimetric data in historical TMLI plans at our institution are summarized at prescription dose levels of 12 Gy and 20 Gy, respectively. Compared to the normal organ dose with a prescription dose of 12 Gy, the mean and median dose to most normal organs at an escalated prescription dose of 20 Gy had an increase less than prescription dose scaling. Dosimetric results from this study can be used as reference data to facilitate clinical implementation of TMLI at other institutions.

## Introduction

Total body irradiation (TBI) is typically used as part of the conditioning regimen for patients undergoing hematopoietic stem cell transplant (1). Traditionally, TBI is delivered using open photon fields at an extended distance from the patient (2). When TBI is given at myeloablative dose levels, shielding of the lungs is necessary to reduce the risk of interstitial pneumonitis. On the other hand, most other body organs receive full dose with conventional TBI treatments. Conventional TBI is associated with numerous acute and long-term complications, among which interstitial pneumonitis is the most common toxicity and contributes to treatment-related mortalities (2). Although previous randomized trials showed that TBI dose escalation reduced post-transplant relapse rate for patients with acute and chronic myeloid leukemia, the therapeutic gain was negated by excessive radiation toxicity with dose escalation (3–6).

To minimize treatment-related toxicities in TBI treatments and to allow for dose escalation, intensity modulated radiotherapy (IMRT) techniques were proposed to deliver radiation dose to targeted sites. Helical tomothearpy was first used to deliver total marrow irradiation (TMI) and total marrow and lymphatic irradiation (TMLI) (7–9). Later, TMI and TMLI were implemented with IMRT fields on conventional medical linear accelerators (linacs) (10–12). Dose escalation clinical trials were carried out at some institutions to evaluate the potential benefits of improvement in therapeutic outcomes with reduced toxicities. Preliminary results from some clinical trials show that TMLI can be safely delivered at an escalated dose of up to 20 Gy without increased rate of extramedullary relapse compared with data using conventional TBI (13–16).

There is growing interest in the radiation oncology community in using the IMRT technique to deliver TMI/TMLI for patients undergoing hematopoietic stem cell transplant. However, TMI/TMLI treatments are currently performed in a small number of institutions and many clinicians lack experience in TMI/TMLI treatment planning. Our group has treated over 400 TMLI patients in the past 17 years under different dose escalation clinical trials and has accumulated many clinical TMLI treatment plans at different dose escalation levels. The purpose of this study is to summarize and present our dosimetric planning experience in TMLI treatment planning to facilitate clinical adoption of this modality by other institutions.

## Materials and Methods

Our institution started TMLI treatments since 2005. Over the years, patient simulation and setup techniques underwent several changes to increase patient comfort and to improve immobilization quality. Current patient setup techniques are presented here.

During CT simulation, the patient is first set up on the CT simulator couch in a head-in supine position. A whole-body vacuum bag extending from the shoulders to the feet is used to immobilize the patient. Both arms are kept straight and close to the body with hands forming loose fists. A thermoplastic head-to-shoulder mask is used to immobilize the head, neck, and shoulder regions, while another thermoplastic mask covers both feet of the patient for immobilization of the lower extremities. Three radiopaque triangulation markers were placed in the abdominal area in the same axial plane to mark the origin of the coordinates used in the CT images. Another set of two radiopaque markers were placed in an axial plane at the upper thigh level to assist with setup of treatment fields for the upper body and lower extremities. A CT simulation is then performed to scan the patient from the top of skull to mid-thigh with an axial slice thickness of 7.5 mm and a field of view that is sufficient to include the patient’s lateral dimension. The patient is asked to maintain normal breathing during the CT simulation. To further evaluate the respiratory motion of internal organs, two additional CT simulations are subsequently taken in the thoracic region with the patient holding breath at the end of normal inspiration and expiration, respectively.

After the upper body CT simulations, the patient is then set up on the couch in a feet-in supine position with the same immobilization devices. A set of three radiopaque triangulation markers are placed at the mid-shin level to mark the origin for the lower-extremity CT simulation. A CT simulation is performed from the lower pelvis to the feet with an axial slice thickness of 7.5 mm and a field of view that is sufficient to include the patient’s lateral dimension.

The upper-body and lower-extremity CT images were sent to a treatment planning system (TPS) (Eclipse, Varian Medical Systems, Palo Alto, California) where the two sets of images were registered based on bony anatomy in the overlapping lower pelvis and upper thigh regions. The CT images taken at the end of inspiration and expiration phases were registered to the CT images with normal breathing based on the same DICOM coordinates. Over 20 normal organ volumes were delineated on the upper body CT simulation images including eyes, lenses, parotids, oral cavity, optic nerves and chiasm, larynx, thyroid, esophagus, lungs (left and right), heart, upper GI, lower GI, kidneys (left and right), bladder, and rectum. For female patients, breasts, ovaries, and uterus are also delineated. For male patients, testes are delineated and, depending on the disease type and treatment protocol, may be treated as one target volume. The upper GI volume includes the stomach and duodenum, while the lower GI volume includes the small and large intestines. Based on CT images at different breathing phases, respiratory motion is included in the contours for certain organs including the esophagus, kidneys, spleen, and liver. Depending on the specific treatment protocol, some or all of the following volumes will be treated: the skeletal bones (excluding the ribs and skull), ribs, skull, spinal canal, lymph nodes, spleen, brain, liver, and testes. To better control dose to some part of the skeletal bone volume, the skull and the ribs are delineated as separate target volumes. The mandible is excluded from the skeletal bone target to better spare adjacent normal organs. To create the planning target volume (PTV) for the skeletal bones, 5- to 10-mm margins are added from the cortical bone surface with larger margins used to the arms, lower extremities, and shoulders. The skeletal bone PTV is then modified to be inside the skin and away from the kidneys and esophagus by at least 5 mm. In addition, no margin is added anteriorly from the vertebrae or into the pelvic cavity to facilitate normal organ sparing. Only outer margins are used for the skull and the rib targets to avoid overlapping with the brain or the lungs.

Historically, TMLI treatments were predominantly given on helical tomotherapy machines at our institution. A jaw size of 5 cm is used for the upper body TMLI treatment plan. The lower extremities are typically treated with three-dimensional conformal radiotherapy (3D-CRT) treatment plans on helical tomotherapy using anterior–posterior (AP) and posterior–anterior (PA) beams.

More recently, TMLI treatments are given with volumetric modulated arc therapy (VMAT) fields on a conventional linac (TrueBeam, Varian Medical Systems, Palo Alto, CA) at our institution. Four to five isocenters are used for the upper body TMLI treatment plan for an adult patient, with two arc fields typically placed for each isocenter. The isocenters are positioned along a longitudinal axis of the patient with no shift in the lateral of anterior–posterior direction. A 120-leaf multi-leaf collimator (MLC) is used to modulate the VMAT fields with a leaf width of 5 mm for the central 40 leaf pairs and a leaf width of 1 cm for the peripheral 20 leaf pairs. The collimator angle is at 90° so that the MLC leaves move along the longitudinal direction of the patient. Asymmetric jaws are used along the patient’s longitudinal direction so that two arc fields at each isocenter cover different patient body lengths. A 6-MV photon beam is used for all the VMAT fields. The lower extremities are treated with static AP/PA photon fields. The upper body VMAT TMLI plan is summed with the lower extremity plans for verification of adequate dose in the junction region at the upper thigh.

Volumetric imaging is used for daily patient setup. On helical tomotherapy, a megavoltage CT (MVCT) or kilovoltage CT (kVCT) scan is performed from the skull to iliac crest. On the conventional linac, two cone-beam CT (CBCT) scans are performed, one in the head and neck region and the other in the abdominal and pelvic region. The average shifts from image registrations of the two CBCT scans with the simulation CT images are used to correct the couch position. After VMAT fields at one isocenter are delivered, the couch is shifted longitudinally to the next isocenter. Orthogonal kV images are taken at the new isocenter for position verification before delivering VMAT fields at the new isocenter.

We started clinical TMLI treatments in 2005. As of now, more than 400 patients received TMLI treatments at different prescription dose levels as dose was escalated in clinical trials, with the prescription dose escalated up to 20 Gy. When prescription dose was escalated to a higher level, treatment plans for the first several patients were optimized to achieve optimal organ sparing. Treatment plans for subsequent patients at this dose level were generated by using normal organ dosimetric results from the first several treatment plans as a reference to evaluate plan quality. In recent years, the dosimetric constraints to the lung volume were updated in our institutional TMLI treatment planning guidelines by requiring the mean lung dose to be less than 8 Gy, based on lung toxicity studies (16, 17).

In this study, treatment plans for the following patient cohorts were retrieved and analyzed:

1. 12-Gy cohort: A dose of 12 Gy was prescribed to the skeletal bones (excluding the ribs and skull), ribs, skull, lymph nodes, spinal canal, and spleen. The brain and liver were normal organs and the dose to them were kept low in plan optimization. The treatment was given in 8 equal fractions with two fractions delivered each day.

2. 20-Gy cohort: A dose of 20 Gy was prescribed to the skeletal bones (excluding the ribs and skull), ribs, skull, lymph nodes, spinal canal, and spleen, while a dose of 12 Gy was prescribed to the liver and brain. The treatment was given in 10 equal fractions with two fractions delivered each day.

A total of 108 patients were found for the 12-Gy cohort while 120 patients were found for the 20-Gy cohort. **Table 1** lists patient characteristics for each cohort. In the 12-Gy cohort, one patient was treated with a VMAT treatment plan on a conventional linac, while other patients were treated with helical tomotherapy. In the 20-Gy cohort, four patients were treated with VMAT treatment plans on a conventional linac, while other patients were treated with helical tomotherapy. Treatments plans using the updated MLD constraint in the 20-Gy cohort were analyzed separately to evaluate the dosimetric impact to the lung and the rib target volume.

**Table 1:** Patient characteristics.

Patient cohort	12-Gy cohort	20-Gy cohort
Number of patients	108	120
Sex (Male/Female)	56/52	64/56
Age/years-old (Range)	54 ± 13 (10–71)	40 ± 12 (17–64)
Prescription dose (Gy)	12	20
Number of fractions	8	10
Dose per fraction (Gy)	1.5	2.0

To facilitate TMLI plan evaluation in our clinical treatment planning workflow, reference dosimetric tables are used. A reference dosimetric table lists representative dosimetric parameters from several previous TMLI plans with the same prescription dose. Such dosimetric parameters include D80 (dose covering 80% of a structure volume), D50 (median dose), and D10 (dose covering 10% of a structure volume) for both targets and normal organs. These parameters were used in our reference dosimetric tables because they are select points on the dose volume histogram (DVH) curves and are representative of the DVH curve shape. To provide reference dosimetric data for institutions that lack clinical TMLI treatment planning experience, we extracted and analyzed dosimetric parameters including D80, D50, mean dose, and D10 from all the historical TMLI treatment plans. The TMLI treatment plan for each patient was retrieved and the treatment plan data containing dose and structure contours were exported as DICOM files. In-house software applications were developed to extract and analyze dosimetric parameters for the targets and normal organs from the DICOM data files. To illustrate the spread of values for each dosimetric parameter, we calculated and presented the 1st quartiles and 3rd quartiles, in addition to the average values, for each dosimetric parameter in each cohort, where the 1st quartile is defined as the middle value between the minimum value and the median value, and the 3rd quartile is defined as the middle value between the maximum value and the median value for a given parameter. Unpaired *t*-tests were used to compare data between different groups of patients, where the result is regarded as statistically significant when the two-tailed *p*-value is less than 0.05. Statistical analysis in this study was performed with a data analysis software system (Excel version 2102, Microsoft Corp., Redmond, WA).

## Results

**Figure 1:**
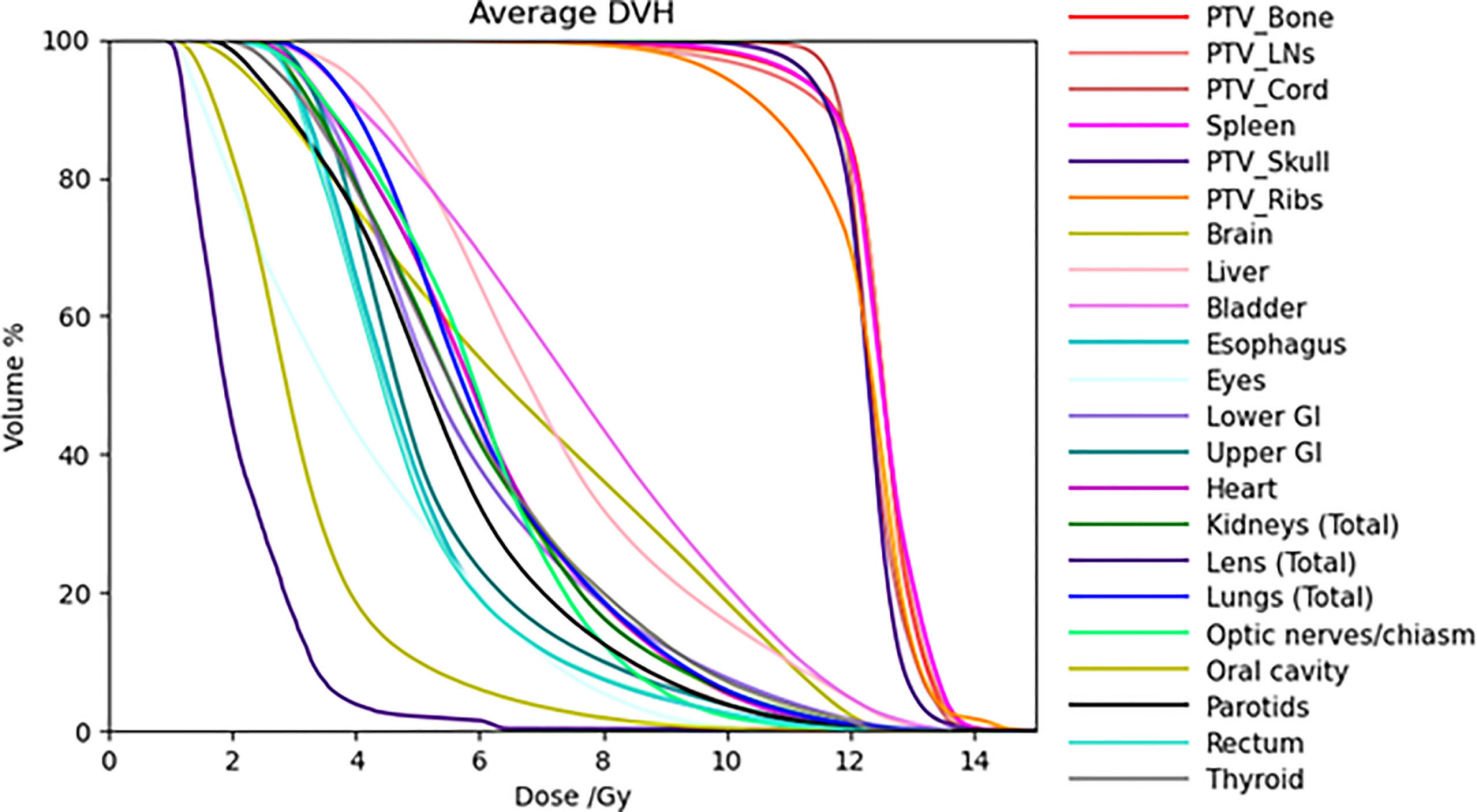
Average DVHs of all the treatment plans for target volumes and most normal organs in the 12-Gy cohort.

**Table 2** lists mean dose and median dose statistics (average, standard deviation, and 1st and 3rd quartiles) for each structure with the 12-Gy cohort. On average, the mean dose for the target volumes ranged from 12.1 Gy to 12.5 Gy and the median dose for the target volumes ranged from 12.3 Gy to 12.6 Gy. Relative to the prescription dose of 12 Gy, the average mean dose for normal organ volumes ranged from 18.3% to 78.3% and the average median dose for normal organ volumes ranged from 18.3% to 77.5%. Among the normal organ structures, the lenses showed the lowest average mean and median dose values while the female breasts showed the highest average mean and median dose values. Of note, the mean lung dose had an average of 6.2 ± 0.6 Gy at this prescription dose level. **Figure 1** shows the average DVH for each structure in the 12-Gy cohort. **Table 3** lists statistics of D80 and D10 for targets and normal organs with the 12-Gy cohort.

**Table 2:** Statistics of mean dose and median dose (D50) for each structure with the 12-Gy cohort.

Structure	Mean dose (Gy)			Median dose (Gy)		
	Avg ± StdDev	1st quartile	3rd quartile	Avg ± StdDev	1st quartile	3rd quartile
Skeletal bones	12.4 ± 0.2	12.3	12.6	12.6 ± 0.3	12.4	12.8
Lymph nodes	12.4 ± 0.3	12.2	12.5	12.6 ± 0.3	12.4	12.8
Spinal canal	12.4 ± 0.4	12.1	12.6	12.4 ± 0.4	12.2	12.6
Spleen	12.5 ± 0.4	12.2	12.7	12.6 ± 0.4	12.4	12.8
Skull	12.3 ± 0.3	12.1	12.4	12.3 ± 0.3	12.2	12.5
Ribs	12.1 ± 0.4	11.9	12.3	12.4 ± 0.4	12.2	12.6
Brain	6.7 ± 0.9	6.0	7.2	6.4 ± 1.1	5.6	7.2
Liver	7.2 ± 0.9	6.6	7.9	6.6 ± 1.1	6.1	7.4
Bladder	7.6 ± 1.5	6.4	8.7	7.2 ± 1.8	5.9	8.6
Female breasts	9.4 ± 1.0	8.8	10.0	9.3 ± 1.1	8.6	10.0
Esophagus	4.9 ± 0.9	4.1	5.5	4.5 ± 0.9	3.7	5.1
Eyes	4.0 ± 1.6	2.5	5.4	3.9 ± 1.7	2.1	5.4
Heart	6.1 ± 1.0	5.5	6.8	5.7 ± 1.1	5.0	6.4
Lower GI	5.9 ± 0.9	5.3	6.3	5.3 ± 1.0	4.7	5.7
Upper GI	5.2 ± 0.8	4.7	5.6	4.7 ± 0.9	4.1	5.0
Kidneys (total)	5.9 ± 1.4	4.8	7.1	5.3 ± 1.4	4.1	6.5
Larynx	5.0 ± 1.4	4.0	5.9	4.4 ± 1.5	3.4	5.3
Lenses (total)	2.2 ± 0.9	1.5	2.7	2.2 ± 1.0	1.4	2.6
Lungs (total)	6.2 ± 0.6	6.0	6.6	5.7 ± 0.4	5.4	6.0
Optic nerves and chiasm	6.0 ± 1.4	4.9	6.9	5.8 ± 1.4	4.7	6.8
Oral cavity	3.2 ± 0.6	2.8	3.5	3.2 ± 0.6	2.8	3.5
Uterus and ovaries	6.4 ± 1.7	5.4	7.3	5.9 ± 1.9	4.4	7.0
Parotids (total)	5.4 ± 1.1	4.6	5.9	4.9 ± 1.3	3.9	5.5
Rectum	4.9 ± 1.0	4.1	5.4	4.3 ± 1.0	3.7	4.8
Thyroid	6.0 ± 1.7	4.8	6.9	5.7 ± 1.8	4.5	6.8

The target volumes are denoted with bold font. The structure of skeletal bones does not include the ribs or skull.

**Table 3:** Statistics of D80 and D10 for each structure with the 12-Gy cohort.

Structure	D80 (Gy)			D10 (Gy)		
	Avg ± StdDev	1st quartile	3rd quartile	Avg ± StdDev	1st quartile	3rd quartile
Skeltal bones	12.2 ± 0.1	12.1	12.3	13.0 ± 0.4	12.7	13.3
Lymph nodes	12.2 ± 0.3	12.0	12.4	13.0 ± 0.4	12.7	13.3
Spinal canal	12.1 ± 0.5	11.9	12.3	12.7 ± 0.4	12.4	13.0
Spleen	12.2 ± 0.4	12.0	12.4	13.0 ± 0.4	12.6	13.3
Skull	12.0 ± 0.3	11.9	12.2	12.7 ± 0.4	12.5	12.8
Ribs	11.5 ± 0.7	11.3	11.8	13.0 ± 0.4	12.7	13.1
Brain	3.7 ± 1.0	3.0	4.4	10.8 ± 0.7	10.3	11.4
Liver	5.4 ± 0.9	4.8	6.0	10.8 ± 1.2	10.3	11.5
Bladder	5.6 ± 1.4	4.2	7.0	10.6 ± 1.5	9.9	11.6
Female breasts	7.7 ± 1.2	6.9	8.5	11.8 ± 0.8	11.3	12.4
Esophagus	3.9 ± 0.7	3.3	4.4	6.9 ± 1.7	5.6	7.8
Eyes	2.8 ± 1.3	1.7	3.7	5.9 ± 2.2	3.7	7.9
Heart	4.6 ± 1.0	3.9	5.4	8.8 ± 1.2	8.2	9.5
Lower GI	4.2 ± 0.9	3.6	4.6	9.1 ± 1.2	8.1	10.0
Upper GI	4.0 ± 0.7	3.4	4.4	7.4 ± 1.6	6.1	8.8
Kidneys (total)	4.7 ± 1.3	3.6	5.6	8.5 ± 1.8	7.2	10.0
Larynx	3.4 ± 1.2	2.6	3.8	7.8 ± 1.9	6.3	9.3
Lens (total)	1.9 ± 0.7	1.3	2.3	2.6 ± 1.4	1.7	3.2
Lungs (total)	4.6 ± 0.7	4.0	5.1	9.0 ± 0.9	8.4	9.6
Optic nerves and chiasm	5.0 ± 1.3	3.9	5.9	7.4 ± 1.8	6.1	8.6
Oral cavity	2.3 ± 0.6	1.9	2.7	5.0 ± 1.2	4.3	5.8
Uterus and ovaries	4.8 ± 1.6	3.6	5.5	9.1 ± 2.3	7.8	10.6
Parotids (total)	4.0 ± 1.2	2.8	4.7	8.0 ± 1.3	7.2	8.7
Rectum	3.9 ± 0.8	3.2	4.4	6.8 ± 1.9	5.4	8.1
Thyroid	4.6 ± 1.7	3.4	5.3	8.2 ± 1.9	6.9	9.7

The target volumes are denoted with bold font. The structure of skeletal bones does not include the ribs or skull.

**Figure 2:**
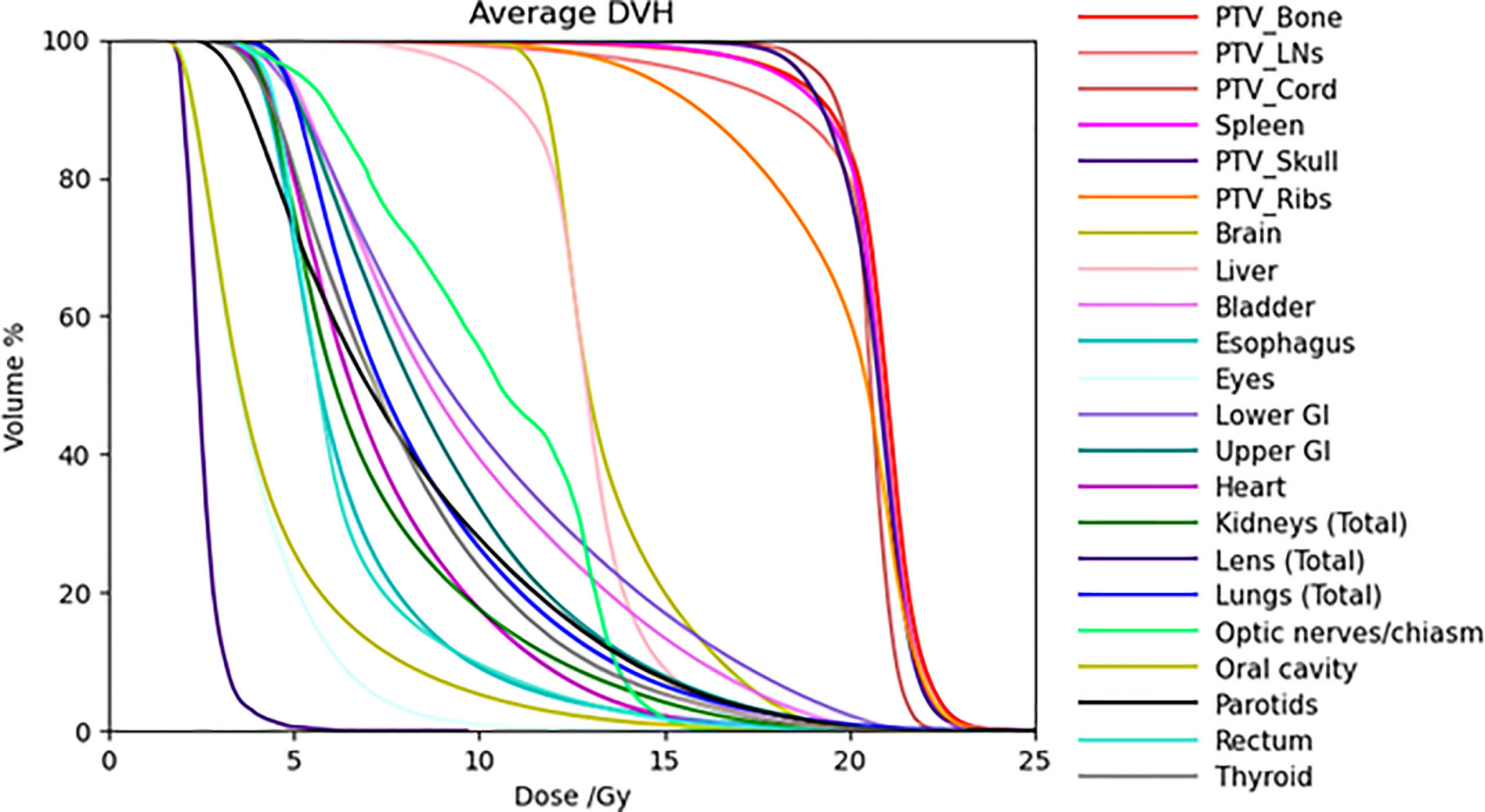
Average DVHs of all the treatment plans for target volumes and some normal organs in the 20-Gy cohort.

**Table 4** lists mean dose and median dose statistics (average, standard deviation, and 1st and 3rd quartiles) for each structure with the 20-Gy cohort. Of note, the brain and liver were prescribed 12 Gy while the other target volumes were prescribed 20 Gy. All the target volumes had a mean dose greater than the prescribed dose except the ribs, which had an average mean dose of 19.6 Gy, while all the target volumes had a median dose greater than the prescribed dose. Relative to the prescription dose of 20 Gy, the average mean dose for the normal organ volumes ranged from 13.0% to 76.0%, and the average median dose for the normal organs ranged from 12.5% to 75.0%. Among the normal organ structures, the lenses showed the lowest average mean and median dose values while the female breasts showed the highest average mean and median dose values. The mean lung dose had an average of 8.5 ± 0.8 Gy for the 20-Gy cohort. **Figure 2** shows the average DVH for each structure in the 20-Gy cohort. **Table 5** lists statistics of D80 and D10 for targets and normal organs with the 20-Gy cohort.

**Table 4:** Statistics of mean dose and median dose (D50) for each structure with the 20-Gy cohort.

Structure	Mean dose (Gy)			Median dose (Gy)		
	Avg ± StdDev	1st quartile	3rd quartile	Avg ± StdDev	1st quartile	3rd quartile
Skeletal bones	20.8 ± 0.4	20.6	21.0	21.1 ± 0.4	20.8	21.3
Lymph nodes	20.4 ± 0.4	20.1	20.6	21.0 ± 0.5	20.7	21.2
Spinal canal	20.5 ± 0.4	20.3	20.7	20.6 ± 0.4	20.4	20.8
Spleen	20.6 ± 0.4	20.4	20.9	20.9 ± 0.5	20.7	21.2
Skull	20.6 ± 0.4	20.4	20.8	20.9 ± 0.4	20.6	21.0
Ribs	19.6 ± 0.8	19.3	20.0	20.4 ± 0.8	20.1	20.9
	19.3 ± 0.8*	18.8*	19.8*	20.2 ± 0.9*	19.9*	20.8*
Brain	13.6 ± 0.7	13.0	14.2	13.0 ± 0.6	12.6	13.4
Liver	12.9 ± 0.6	12.5	13.2	13.0 ± 0.6	12.6	13.3
Bladder	9.7 ± 1.5	8.7	10.6	8.6 ± 1.8	7.5	9.7
Female breasts	15.2 ± 1.6	14.3	16.1	15.0 ± 1.9	13.9	16.0
Esophagus	6.5 ± 0.9	6.1	6.9	5.7 ± 0.7	5.4	6.0
Eyes	4.0 ± 0.8	3.4	4.3	3.6 ± 0.8	3.1	3.9
Heart	7.4 ± 0.6	7.0	7.8	6.5 ± 0.6	6.1	6.9
Lower GI	10.2 ± 1.1	9.5	10.8	9.0 ± 1.3	8.2	9.6
Upper GI	9.0 ± 1.4	8.1	9.9	8.0 ± 1.5	6.9	8.9
Kidneys (total)	7.3 ± 0.7	6.8	7.8	6.0 ± 0.7	5.5	6.4
Larynx	7.5 ± 2.2	6.1	8.7	6.6 ± 2.4	4.9	8.2
Lenses (total)	2.6 ± 0.4	2.3	2.7	2.5 ± 0.4	2.2	2.6
Lungs (total)	8.5 ± 0.8	7.9	9.1	7.4 ± 0.6	6.8	7.9
	7.8 ± 0.4*	7.7*	7.9*	6.7 ± 0.4*	6.4*	7.0*
Optic nerves and chiasm	10.2 ± 1.9	8.8	11.3	10.3 ± 2.5	8.4	12.5
Oral cavity	4.4 ± 1.0	3.7	4.9	3.6 ± 0.8	3.0	4.0
Uterus and ovaries	8.6 ± 2.1	7.6	9.8	7.6 ± 2.1	6.1	8.5
Parotids (total)	8.1 ± 1.3	7.2	8.8	7.0 ± 1.5	5.9	7.8
Rectum	6.5 ± 0.9	5.9	6.9	5.5 ± 0.8	5.0	6.0
Thyroid	8.0 ± 2.1	6.8	8.6	7.4 ± 2.3	6.2	8.0

The target volumes are denoted with bold font. The structure of skeletal bones does not include the ribs or skull.^*^Dosimetric data after the updated mean lung dose criteria was used in treatment planning.

**Table 5:** Statistics of D80 and D10 for each structure with the 20-Gy cohort.

Structure	D80 (Gy)			D10 (Gy)		
	Avg ± StdDev	1st quartile	3rd quartile	Avg ± StdDev	1st quartile	3rd quartile
**Skeletal bones**	20.3 ± 0.3	20.3	20.5	21.7 ± 0.5	21.4	22.0
Lymph nodes	20.0 ± 0.6	19.8	20.4	21.6 ± 0.5	21.3	21.9
Spinal canal	20.2 ± 0.4	20.1	20.4	21.0 ± 0.5	20.7	21.3
Spleen	20.2 ± 0.5	20.0	20.5	21.6 ± 0.6	21.2	21.9
Skull	19.9 ± 0.5	19.7	20.2	21.6 ± 0.6	21.3	21.8
Ribs	17.9 ± 1.3	17.4	18.8	21.6 ± 0.6	21.3	21.8
Brain	12.2 ± 0.4	12.0	12.4	16.2 ± 1.5	14.7	17.6
Liver	12.1 ± 0.4	11.9	12.3	14.7 ± 1.3	13.7	15.4
Bladder	6.4 ± 1.3	5.4	7.0	15.4 ± 2.1	14.2	16.8
Female breasts	12.2 ± 1.9	11.0	13.1	19.8 ± 1.4	19.3	20.8
Esophagus	4.8 ± 0.6	4.4	5.1	9.6 ± 2.0	8.4	10.7
Eyes	2.7 ± 0.5	2.4	2.9	6.0 ± 1.6	4.9	6.6
Heart	5.0 ± 0.6	4.7	5.4	11.5 ± 1.1	10.8	12.2
Lower GI	6.3 ± 1.2	5.5	7.0	16.7 ± 1.5	15.8	17.6
Upper GI	6.3 ± 1.4	5.3	7.2	13.8 ± 1.9	12.7	15.1
Kidneys (total)	4.9 ± 0.6	4.5	5.3	12.0 ± 1.6	11.0	12.9
Larynx	4.7 ± 1.7	3.6	5.2	12.2 ± 3.1	10.2	14.2
Lens (total)	2.3 ± 0.3	2.1	2.4	2.9 ± 0.6	2.5	3.2
Lungs (total)	5.6 ± 0.6	5.3	6.0	13.3 ± 1.6	12.1	14.5
Optic nerves and chiasm	8.3 ± 2.5	6.7	9.9	13.0 ± 1.6	12.5	14.0
Oral cavity	2.8 ± 0.6	2.4	3.2	7.8 ± 2.4	5.8	9.1
Uterus and ovaries	5.8 ± 1.3	4.9	6.4	13.7 ± 3.8	11.3	16.2
Parotids (total)	4.7 ± 1.1	4.0	5.2	13.8 ± 2.0	12.3	15.0
Rectum	5.0 ± 0.7	4.6	5.5	9.5 ± 2.2	7.9	11.0
Thyroid	5.7 ± 1.9	4.5	5.8	11.9 ± 2.7	10.0	13.4

The target volumes are denoted with bold font. The structure of skeletal bones does not include the ribs or skull.

**Figure 3:**
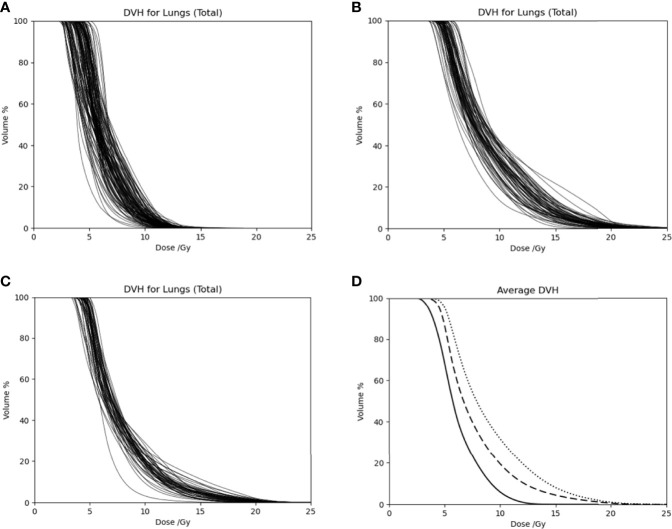
Dose volume histograms of the total lung for **(A)** patients in the 12-Gy cohort, **(B)** patients in the 20-Gy cohort before the updated mean lung dose constraint was used, and **(C)** patients in the 20-Gy cohort after the updated mean lung dose constraint was used. **(D)** Average DVH curves for patients in the 12-Gy cohort (solid curve), patients in the 20-Gy cohort before the updated mean lung dose constraint was used (dotted curve), and patients in the 20-Gy cohort after the mean lung dose constraint was used (dashed curve).

**Table 4** also lists dose statistics for the total lung and the ribs target volume when only the cases after clinical implementation of the updated mean lung dose constraint in 2018 were included. The mean lung dose had an average of 7.8 ± 0.4 Gy while the rib target volume had an average of 19.3 ± 0.8 Gy. Unpaired *t*-tests were performed on the mean lung dose and mean rib target dose between the treatment plans in this 20-Gy cohort before 2018 and those after 2018, and the results showed a statistically significant difference (two-tailed *p*-value < 0.01 for both structures). To illustrate the difference in the lung dose distributions, **Figure 3** shows the DVHs of the total lung for patients in the 12-Gy cohort, patients in the 20-Gy cohort before the updated mean lung dose constraint was used in treatment planning, and patients in the 20-Gy cohort after the updated mean lung dose constraint was used, respectively. Average lung DVH curves are also shown in **Figure 3D** for each of the three groups of patients.

**Figure 4:**
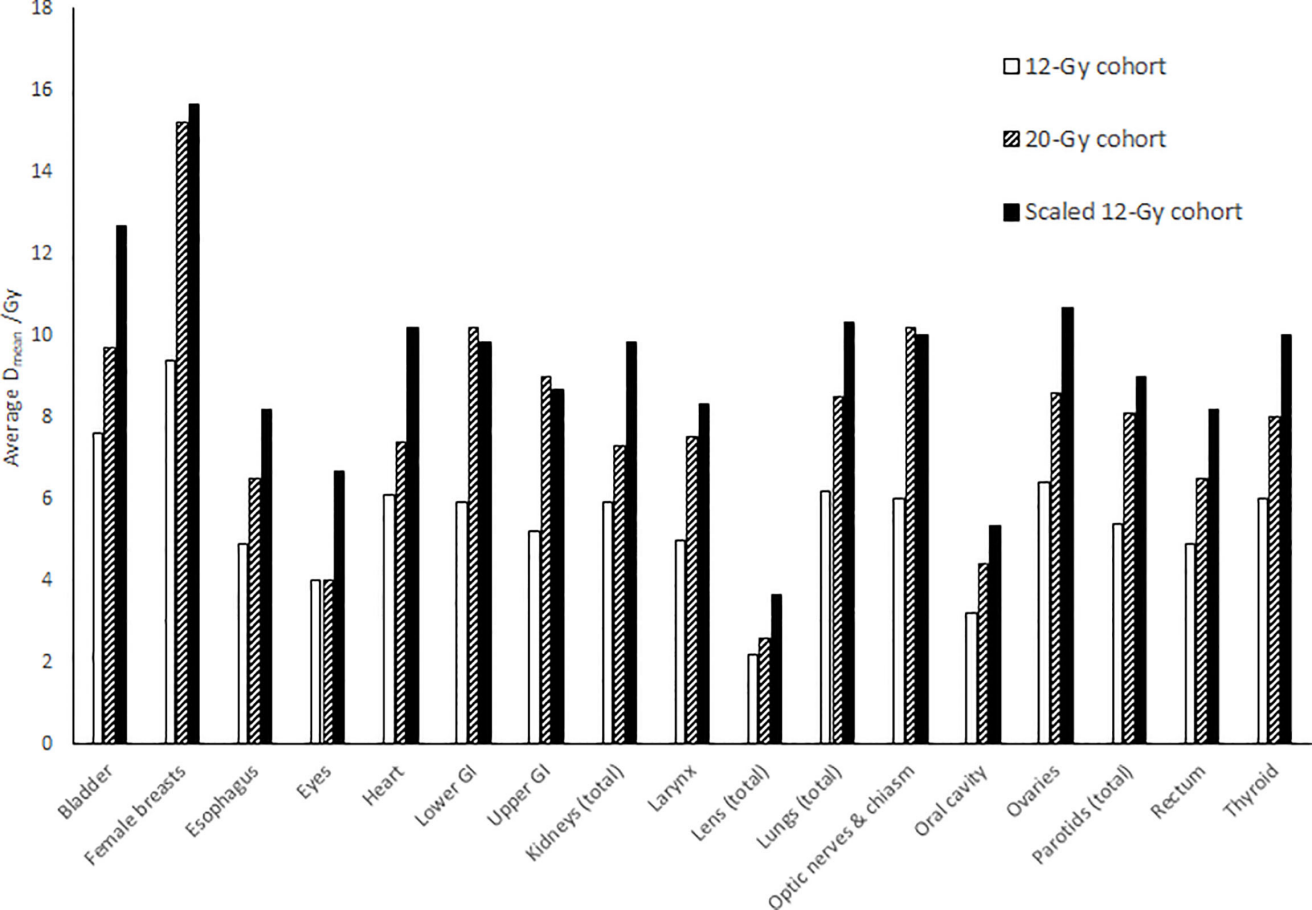
Comparison of average mean dose (D_mean_) to each normal organ between the 12-Gy cohort and the 20-Gy cohort. The “Scaled 12-Gy cohort” data series shows average D_mean_ for the 12-Gy cohort scaled by the prescription dose ratio (20/12).

**Figure 4** compares average mean dose to each normal organ volumes between the 12-Gy cohort and the 20-Gy cohort. Compared to the average mean dose in the 12-Gy cohort plans, the average mean dose in the 20-Gy cohort plans had an increase ranging from 0.0% to 73.1%, while the prescription dose increased by 66.7% from 12 Gy to 20 Gy. Compared to the average median dose in the 12-Gy cohort plans, the average median dose for the normal organ volumes had an increase/decrease ranging from −7.7% to 77.6%. Four normal organ volumes showed more than 50% increase in both the average mean dose and median dose (female breasts, lower GI, upper GI, and optic nerves and chiasm), and three normal organ volumes (lower GI, upper GI, and optic nerves and chiasm) showed higher average mean dose in the 20-Gy cohort than the average mean dose 12-Gy cohort scaled by the prescription dose ratio of 20/12.

**Figure 5:**
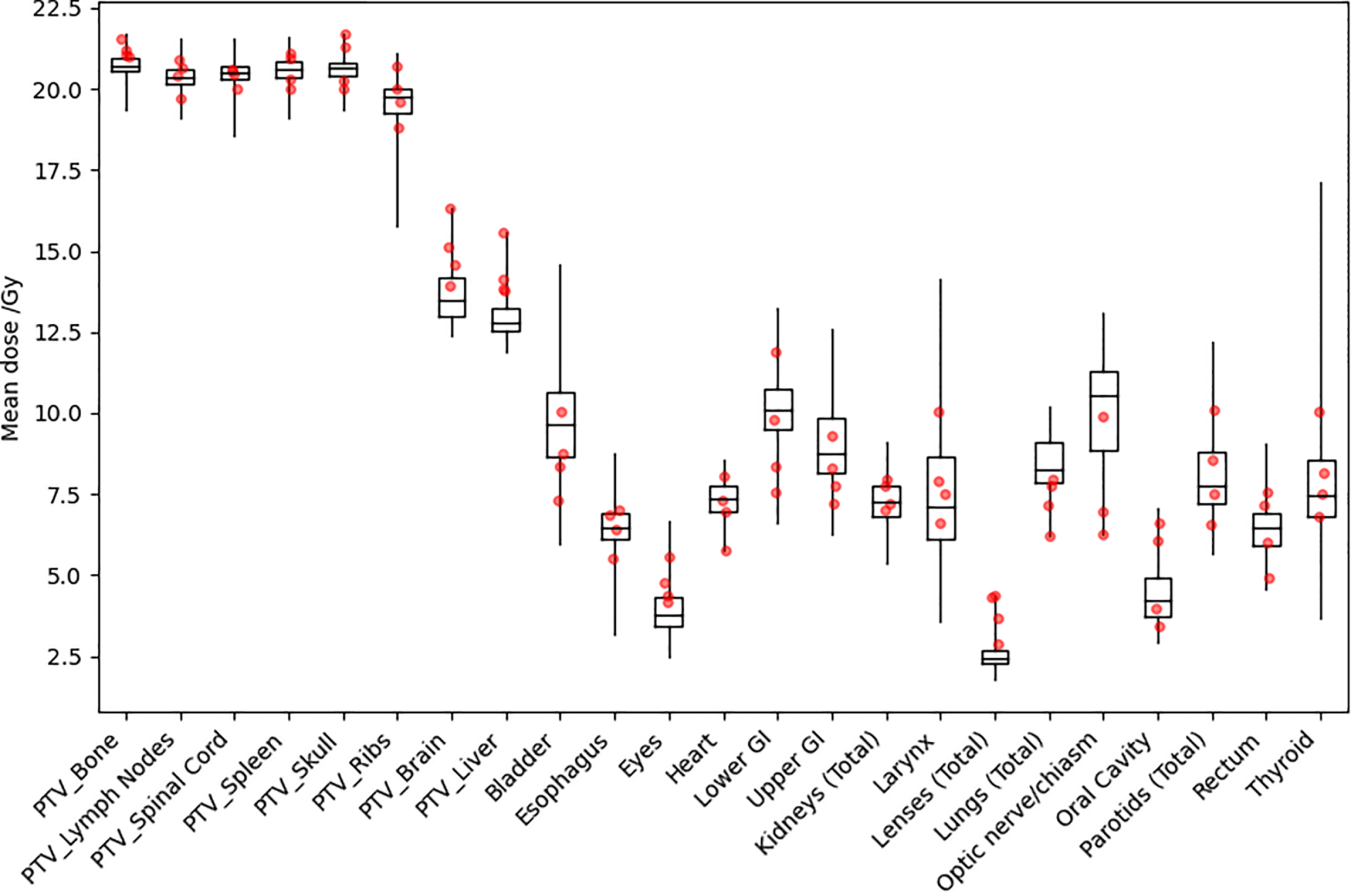
Distribution of mean dose (Dmean) for target volumes and major normal organs in the 20-Gy cohort. The median value of Dmean for each structure is shown at the horizontal bar in the middle of each rectangle. The 1st and 3rd quartiles are shown as the lower and upper horizontal sides of each rectangle. The minimum and maximum range of Dmean is shown as the vertical lines extending from each rectangle. The red dots are Dmean data in the four VMAT TMLI treatment plans in the 20-Gy cohort.

**Figure 5** shows distributions of mean dose for target volumes and major normal organ volumes in historical TMLI treatment plans for the 20-Gy cohort. The minimum, maximum, and first, second, and third quartiles of the mean dose are shown in the box plot for each target and each normal organ. For comparison, the mean dose data to targets and normal organ volumes in the four VMAT TMLI treatment plans in the 20-Gy cohort are also included in **Figure 5**.

## Discussion

This study presents a comprehensive summary of dosimetric plan data in clinical TMLI treatment plans at two prescription dose levels, one at the standard myeloablative dose level of 12 Gy and the other at the dose escalation level of 20 Gy. This study includes the largest TMLI patient cohorts to date. Representative dosimetric data in the historical TMLI treatment plans are provided, which can be used as reference data to facilitate clinical implementation of TMLI at other institutions.

Wong et al. evaluated dosimetric feasibility of dose escalation with TMI or TMLI up to 20 Gy in an early study (8). However, in that study, only the bone marrow volume was escalated to 20 Gy while 12 Gy was prescribed to the lymphatic volume, skull, and ribs. This current study showed practically achievable normal organ sparing in clinical TMLI treatment plans at the 20-Gy prescription dose level given to the lymphatic volume, skull, and ribs. **Table 5** shows that most of the normal organs had less than 50% increase in mean and median dose in the 20-Gy cohort treatment plans compared to the 12-Gy cohort treatment plans, despite an increase of 66.7% from 12 Gy to 20 Gy for most target volumes and inclusion of the brain and liver as target volumes. Only four organ volumes showed more than 50% increase in mean and median dose. Of note, inclusion of the brain and liver as target volumes in the 20-Gy dose escalation protocols negatively affected sparing of some normal organs. The optic nerves and chiasm had more than 50% increase because it is partially surrounded by the brain and skull, while the upper GI and lower GI had more than 50% increase partly because of the proximity to the liver. The female breasts also showed more than 50% increase in median and mean dose due to proximity to the ribs.

**Tables 2**–**5** show that dose to the ribs was lower compared to other target volumes as dose to the ribs was negatively affected by sparing of the lungs, as we prioritized lung sparing rather than dose coverage to the ribs. In addition, **Table 4** shows that dose to the ribs was lower in those plans with the updated MLD criteria. Of note, even with the updated MLD criteria in treatment planning, the ribs still received escalated dose with an average median dose of 19.3 Gy and an average mean dose of 20.2 Gy. Dosimetric consistency of the ribs was also affected by sparing of the lungs. **Table 4** shows that dose to the ribs had greater variation compared to other target volumes. Based on our clinical planning experience, the rib volume is the most challenging target volumes to achieve dosimetric consistency in TMLI treatment planning. Further improvement in treatment planning techniques is needed to deliver consistent dose to the ribs.

Clinical TMLI treatments using VMAT fields started in 2021 at our institution. We have delivered TMLI treatments using VMAT fields on conventional linacs for more than ten patients with prescription dose ranging from 12 Gy to 20 Gy. In this study, the dosimetry data for those VMAT TMLI plans with a prescription dose of 20 Gy were presented. **Figure 5** shows that dose in the VMAT TMLI plans fell within the range in historical TMLI plans for most normal organs. Treatment planning and delivery using VMAT fields present unique challenges. Due to field size limits, multiple isocenters are needed and adjacent fields need to overlap to ensure adequate target dose coverage. In an image-guided patient setup, multiple image acquisitions are needed at different isocenters due to limitations in CBCT image size in the longitudinal direction. On the other hand, since conventional linacs are more widely available, this technique provides access for more patients receiving TMLI treatments. Therefore, we are actively making improvement in treatment planning and delivery efficiency in VMAT-based TMLI and plan to present our treatment planning and delivery experience in a separate report.

## Conclusions

Dosimetric data in historical TMLI plans at our institution are summarized at prescription dose levels of 12 Gy and 20 Gy, respectively. Compared to the normal organ dose with a prescription dose of 12 Gy, the mean and median dose to most normal organs at an escalated prescription dose of 20 Gy had an increase less than prescription dose scaling. The VMAT TMLI plans achieved normal organ dose sparing within the range of historical TMLI plans. Dosimetric results from this study can be used as reference data to facilitate clinical implementation of TMLI at other institutions.

## Data Availability Statement

The original contributions presented in the study are included in the article/supplementary material. Further inquiries can be directed to the corresponding author.

## Author Contributions

CH performed data collection, data analysis, and manuscript preparation. AL participated in the design of this study and performed critical review of the manuscript. JW participated in the design of this study, creation of methods for this study, and data analysis, and performed critical review of the manuscript. All authors contributed to the article and approved the submitted version.

## Funding

This research was partially supported by funding provided by Accuray, Inc and Varian Medical Systems, Inc. The funder Varian Medical Systems, Inc. was not involved in the study design, collection, analysis, interpretation of data, the writing of this article or the decision to submit it for publication.

## Conflict of Interest

This study received funding from Accuray, Inc. The funder had the following involvement with the study: Part of the fund was used to support establishment of a total marrow irradiation patient registry at our institution. All authors declare no other competing interests.

## Publisher’s Note

All claims expressed in this article are solely those of the authors and do not necessarily represent those of their affiliated organizations, or those of the publisher, the editors and the reviewers. Any product that may be evaluated in this article, or claim that may be made by its manufacturer, is not guaranteed or endorsed by the publisher.
